# Inter-replicon Gene Flow Contributes to Transcriptional Integration in the *Sinorhizobium meliloti* Multipartite Genome

**DOI:** 10.1534/g3.117.300405

**Published:** 2018-03-21

**Authors:** George C. diCenzo, Deelaka Wellappili, G. Brian Golding, Turlough M. Finan

**Affiliations:** Department of Biology, McMaster University, Hamilton, Ontario, Canada L8S 4K1

**Keywords:** transcriptomics, genome evolution, genome reduction, horizontal gene transfer, hydroxybutyrate

## Abstract

Integration of newly acquired genes into existing regulatory networks is necessary for successful horizontal gene transfer (HGT). Ten percent of bacterial species contain at least two DNA replicons over 300 kilobases in size, with the secondary replicons derived predominately through HGT. The *Sinorhizobium meliloti* genome is split between a 3.7 Mb chromosome, a 1.7 Mb chromid consisting largely of genes acquired through ancient HGT, and a 1.4 Mb megaplasmid consisting primarily of recently acquired genes. Here, RNA-sequencing is used to examine the transcriptional consequences of massive, synthetic genome reduction produced through the removal of the megaplasmid and/or the chromid. Removal of the pSymA megaplasmid influenced the transcription of only six genes. In contrast, removal of the chromid influenced expression of ∼8% of chromosomal genes and ∼4% of megaplasmid genes. This was mediated in part by the loss of the ETR DNA region whose presence on pSymB is due to a translocation from the chromosome. No obvious functional bias among the up-regulated genes was detected, although genes with putative homologs on the chromid were enriched. Down-regulated genes were enriched in motility and sensory transduction pathways. Four transcripts were examined further, and in each case the transcriptional change could be traced to loss of specific pSymB regions. In particularly, a chromosomal transporter was induced due to deletion of *bdhA* likely mediated through 3-hydroxybutyrate accumulation. These data provide new insights into the evolution of the multipartite bacterial genome, and more generally into the integration of horizontally acquired genes into the transcriptome.

Horizontal gene transfer (HGT) is pervasive in bacterial species and plays a significant role in the genotypic and phenotypic diversity between two isolates of the same species or genus (Ochman *et al.* 2000). The term ‘pan-genome’ is used to describe all genes present in all strains of a particular species or genus, with the ‘core genome’ referring to genes conserved in all strains and the ‘accessory genome’ used in reference to genes absent in at least one strain (Tettelin *et al.* 2005). The pan-genome of 33 *Sinorhizobium meliloti* strains was determined to consist of 21,000 genes of which 78% were variably present or absent (Sugawara *et al.* 2013). In the case of the genus *Prochlorococcus*, the genome size is a mere 2,000 genes in comparison to a pan-genome size of at least 58,000 genes (Baumdicker *et al.* 2012). Most genes acquired through HGT are rapidly lost from the genome (Lawrence and Ochman 1998; Hao and Golding 2006). Many factors influence whether the maintenance of a gene acquired through HGT is selected for, among which are that the gene must be appropriately expressed and regulated, and that it does not interfere with the expression or activity of existing gene products (Pál, Papp, and Lercher 2005b; Sorek *et al.* 2007; Amorós-Moya *et al.* 2010; Cohen *et al.* 2011; Park and Zhang 2012; Baltrus 2013; San Millan *et al.* 2015). *In silico* analyses have predicted that newly acquired genes are poorly integrated into existing regulatory networks, but that these genes are eventually integrated into the networks (Lercher and Pál 2008). However, to date there has been little experimental analysis of the effects of large-scale horizontal gene transfer on the existing transcriptome of the host cell.

Approximately 10% of bacterial species contain a multipartite genome consisting of at least two DNA replicons of 300 kb or larger (diCenzo and Finan 2017). In these genomes, there is a primary chromosome that contains most if not all core cellular processes and is primarily vertically transmitted (Harrison *et al.* 2010; Galardini *et al.* 2013). The secondary replicons can be broadly classified as megaplasmids or chromids. Megaplasmids originate through recent HGT and are continuously undergoing rapid gene gain and gene loss (Guo *et al.* 2009; Cooper *et al.* 2010; Galardini *et al.* 2013). As such, the characteristics of entire megaplasmids largely mimics those of individual genes acquired through recent HGT. Chromids can be thought of as domesticated megaplasmids that are largely derived from ancient HGT (Wong and Golding 2003; Harrison *et al.* 2010; Galardini *et al.* 2013). These replicons therefore display many of the same characteristics as individual genes obtained through ancient HGT events. In addition, chromids encode a few core processes as a result of inter-replicon gene flow from the chromosome (Harrison *et al.* 2010). A clear example of this is in the genus *Sinorhizobium*, where the translocation of a contiguous 69 kb region from the chromosome to the chromid resulted in the transfer of two genes essential for cell viability (diCenzo *et al.* 2013; diCenzo *et al.* 2016b). Studying the features of secondary replicons and how the cell responds to the gain or loss of these replicons can serve as a tool to study the effects of large-scale HGT and how the time since acquisition of horizontally transferred genes influences their properties.

In some ways, secondary replicons function as separate entities from the chromosome. Transcriptional studies with *S. meliloti*, *Burkholderia cenocepacia*, *Vibrio cholerae*, *Rhizobium leguminosarum*, and *Rhizobium phaseoli* indicated that genes from particular replicons were over-represented among the genes differentially expressed in response to adaptation to certain niches (Xu *et al.* 2003; Becker *et al.* 2004; Barnett *et al.* 2004; Yoder-Himes *et al.* 2010; Ramachandran *et al.* 2011; López-Guerrero *et al.* 2012). *In silico* metabolic modeling work with *S. meliloti* predicted that the chromid had a much greater contribution to fitness in the rhizosphere than in bulk soil (diCenzo *et al.* 2016a). An *in silico* analysis of regulons in *S. meliloti* predicted that most transcription factors regulate genes on their own replicon (Galardini *et al.* 2015).

On the other hand, inter-replicon co-operation does occur, and the level of integration appears dependent on how long the replicons have co-existed within the same cell. Despite occupying distinct territories of the nucleoid, there are physical interactions between the two replicons of *V. cholerae* (Galli *et al.* 2016) and initiation of chromid replication is dependent on a chromosomally encoded factor (Baek and Chattoraj 2014; Val *et al.* 2016). Elimination of the chromid from *S. meliloti* resulted in a major perturbation of the intra-cellular and extra-cellular metabolomics profile unlike elimination of the megaplasmid (Fei *et al.* 2016), although many changes may have been indirect effects of the impaired growth. An *in silico* analysis predicted functional links between each of the seven replicons of *Rhizobium etli*, with the two most recently acquired replicons having the least connections (González *et al.* 2006). Moreover, experimental and *in silico* studies suggest a minority of *S. meliloti* transcription factors regulate genes on other replicons, with greater cross-talk between the two older replicons (Pini *et al.* 2015; Galardini *et al.* 2015). Similarly, the chromosomally encoded RpoS regulates expression of both chromosomal and chromid genes in *V. cholerae* (Heidelberg *et al.* 2000). However, it has not been experimentally examined on a global scale the level of transcriptional integration of each replicon in a multipartite genome.

The genome of *S. meliloti* is split between a 3.65 Mb chromosome, a 1.68 Mb pSymB chromid, and a 1.35 Mb pSymA megaplasmid (Galibert *et al.* 2001), and derivatives that lack all of pSymA, pSymB, or both (a 45% genome reduction) have been constructed (Oresnik *et al.* 2000; diCenzo *et al.* 2014). Here, we use RNA sequencing to examine the transcriptional consequences of the loss of the chromid and/or the megaplasmid. Our data indicate that extended evolution of a chromosome and a secondary replicon in the same cell results in significant integration at the transcriptional level, and that this is partly driven by inter-replicon gene flow. These results provide novel insights into the evolution of multipartite genomes as well as into the integration of horizontally acquired genes into the genome.

## Materials and Methods

### Growth conditions

Antibiotic concentrations, growth conditions for *S. meliloti* and *E. coli*, and the compositions of LB and LBmc were as described previously (diCenzo *et al.* 2014). LB and LBmc were supplemented with 2 µM CoCl_2_ for growth of *S. meliloti* strains (Cheng *et al.* 2011). M9-sucrose minimal medium consisted of: 41 mM Na_2_HPO_4_, 22 mM KH_2_PO_4_, 8.6 mM NaCl, 18.7 mM NH_4_Cl, 41 µM biotin, 42 nM CoCl_2_, 1 mM MgSO_4_, 0.25 mM CaCl_2_, 38 µM FeCl_3_, 5 µM thiamine-HCl, and 10 mM sucrose. When included, DL-3-hydroxybutyrate was added to a final concentration of 10 mM.

### Genetic manipulations and bacterial strains

DNA manipulations and recombinant techniques, bacterial matings, and ΦM12 transductions were performed as described before (Finan *et al.* 1984; Sambrook *et al.* 1989; Cowie *et al.* 2006; Milunovic *et al.* 2014). Recombinant plasmids were produced through sequence and ligation independent cloning (Jeong *et al.* 2012). All bacterial strains and plasmids are listed in Table S1, and oligonucleotides are listed in Table S2.

All strains used for RNA-seq analysis were constructed previously and are listed in Table 1 (diCenzo *et al.* 2014; diCenzo *et al.* 2016b). Briefly, the two essential genes of pSymB (tRNA^arg^ and *engA*) were integrated into the chromosome of a *S. meliloti* strain lacking pSymA, following which pSymB was removed to produce the strain ∆pSymAB (RmP3496) (diCenzo *et al.* 2014). The pSymA megaplasmid was conjugated into this strain to produce ∆pSymB (RmP3497) (diCenzo *et al.* 2016b). Similarly, ∆pSymA and the ‘wild type’ strains were produced by conjugating either pSymB or both pSymA and pSymB into the ∆pSymAB strain (diCenzo *et al.* 2016b). Finally, ∆pSymAB_ΩNGR69_ was constructed by integrating the 69 kb ETR region from *Sinorhizobium fredii* NGR234 into the chromosome of a *S. meliloti* strain lacking pSymA, followed by the removal of pSymB (diCenzo *et al.* 2016b). The ETR region (standing for *engA*-tRNA^arg^-*rmlC*) refers to a 69 kb region that translocated from the chromosome to pSymB in a *S. meliloti* ancestor (diCenzo *et al.* 2013).

**Table 1:** Description of the strains used in the RNA-seq experiment

Strain	Identifier	Genotype	Reference
∆pSymAB	RmP3496	A Rm2011 derivative with the two essential pSymB genes (engA and tRNAarg) in the chromosome, and lacking the pSymA megaplasmid and the pSymB chromid.	(diCenzo *et al.* 2014)
∆pSymB	RmP3497	∆pSymAB with the pSymA megaplasmid re-introduced into the genome	(diCenzo *et al.* 2016b)
∆pSymA	RmP3498	∆pSymAB with the pSymB chromid re-introduced into the genome	(diCenzo *et al.* 2016b)
Wild type	RmP3499	∆pSymAB with the pSymA and pSymB replicons both re-introduced into the genome	(diCenzo *et al.* 2016b)
∆pSymAB_ΩNGR69_	RmP3500	A Rm2011 derivative containing the entire ETR (*engA*-tRNA-*rmlC*) region of *S. fredii* NGR234 integrated into the chromosome, and lacking the pSymA megaplasmid and the pSymB chromid.	(diCenzo *et al.* 2016b)

### RNA sequencing and analysis

Cell pellets from biological duplicates of each of the five strains (∆pSymAB, ∆pSymB, ∆pSymA, wild type, ∆pSymAB_ΩNGR69_) grown in 5 mL of M9-sucrose were washed with fresh M9-sucrose and used to inoculate 100 mL M9-sucrose cultures to a starting OD_600_ ∼0.1. Cultures were grown at 30° with shaking until an OD_600_ ∼0.7 was reached, at which point 45 mL of cultures was immediately mixed with 5 mL of ice-cold cell stop solution (5% unbuffered phenol, 95% ethanol). Cells were pelleted by centrifugation at 3,700 *g* at 4° for 10 min, and the pellets flash frozen with liquid nitrogen. Frozen pellets were stored at -80° until use.

Isolation of total RNA from each pellet, as well as the confirmation of RNA integrity and lack of DNA contamination, was performed as described previously (diCenzo *et al.* 2017). Depletion of the rRNA with Ribo-Zero rRNA Removal kits (Illumina), cDNA library synthesis, and cDNA sequencing using an Illumina HiSequation 1500, with 60 bp single reads, was performed at the Farncombe Family Digestive Health Research Institute located at McMaster University.

As a quality control step, FastQC (Andrews 2010) was run on all sequencing samples. Reads were trimmed with cutadapt (Martin 2011) with options to ensure quality of 25 from both ends, minimum size of 20 nts, and no Ns at the ends. Each replicon of the *S. meliloti* Rm2011 genome (Sallet *et al.* 2013) were individually indexed with Burrows-Wheeler Aligner (bwa) (Li and Durbin 2009). The bwa algorithm was then used to index and map the reads to each replicon, and htseq-count (Anders *et al.* 2015) was used to count the number of reads to each annotated gene. Differential expression analysis was finally performed using DESeq2 (Love *et al.* 2014), with the DESeq2 analysis performed independently for each of the three replicons. The complete set of results from the DESeq2 analysis is summarized in File S2, File S3, and File S4, and the raw sequencing reads are available online through the Gene Expression Omnibus (GEO accession: GSE106129).

### Computational analyses

Heatmaps were generated with the heatmap.2 function in the gplots package of R (Warnes *et al.* 2009), and the clustering performed using average linkage with a Pearson correlation distance. Operon prediction was performed with the Rockhopper software (Tjaden 2015) using the raw RNA-seq data generated in this study (File S5). Genes were classified as core, accessory, or dispensable based on a previously published pangenome analysis (Galardini *et al.* 2015). The Blast 2.6.0+ software (Camacho *et al.* 2009) was used to search the entire *S. meliloti* Rm2011 chromosomal proteome (NC_003047) against the pSymA proteome (NC_003037) and pSymB proteome (NC_003078) (Sallet *et al.* 2013). Putative homologs were identified as those that shared at least 30% identity over at least 60% of the query protein. All chromosomal genes were functionally annotated with Gene Ontology (GO) terms, Cluster of Orthologous Genes (COG) categories, and KEGG pathway terms using the eggNOG webserver (Ashburner *et al.* 2000; Tatusov *et al.* 2003; Huerta-Cepas *et al.* 2016; Kanehisa *et al.* 2016). Custom perl scripts were used to summarize the functional annotation data, and functionally similar GO terms were grouped using the REVIGO webserver (Supek *et al.* 2011).

### Reporter enzyme assays

Biological triplicates of each strain of interest were grown in either 5 mL M9-sucrose test tube cultures or in 200 µL M9-sucrose cultures in 96-well microtitre plates. β-Galactosidase and β-glucuronidase assays were performed in 96-well microtitre plates as described elsewhere (Cowie *et al.* 2006). The sole change to the procedure was that 10 µL of culture, 40 µL of reaction buffer, and 50 µL of stop buffer was used per reaction. Control reactions were prepared for each culture by mixing 40 µL start buffer and 50 µL stop buffer prior to the addition of 10 µL of culture. The A_420_ (β-galactosidase) or A_405_ (β-glucuronidase) of each reaction and control reaction, and the OD_600_ of each culture, was measured using a Biotek Cytation3. Miller Units were calculated following the subtraction of the A_420_/A_405_ values of the control reaction from the A_420_/A_405_ reading of the true reaction.

### Confirmation of RNA-seq expression data

For confirmation of the RNA-seq data, *lacZ* or *gusA* transcriptional fusions to ten genes of interest were identified in the *S. meliloti* fusion library (Cowie *et al.* 2006). The fusion library consists of a collection of *S. meliloti* strains with pTH1522 plasmid single cross-over recombinants integrated at known locations throughout the *S. meliloti* genome (http://info.mcmaster.ca/fusionlibrary/sinoseq.php). The fusion strains used in this study were: SmFL55 (*smc02350*^+^::*lacZ*), SmFL107 (*smc01946*^+^::*gusA*), SmFL1109 (*sma2085*^+^::*lacZ*, *sma2087*::*gusA*), SmFL1435 (*smc03027*^+^::*lacZ*), SmFL1485 (*sma0089*^+^::*gusA*), SmFL1705 (*smc03133*^-^::*gusA*), SmFL4407 (*smc03121*^+^::*lacZ*), SmFL6033 (*smc02780*^+^::*gusA*), and SmFL6082 (*smb21039*^-^::*lacZ*). Each fusion was recombined via transduction from the appropriate fusion library stain into each of the five strains used in the RNA-seq analysis, selecting for the gain of gentamicin resistance.

### Transcriptional changes in pSymB deletion mutant strains

To identify pSymB gene regions that modulate expression of four genes of interest (*smc02350*, *smc03121*, *sma2085*, and *sma2087*), the appropriate gene fusions were combined with the following eleven *S. meliloti* strains with large, defined deletions of pSymB (Milunovic *et al.* 2014): ∆B154 (pSymB nt. 62,137-100,636), ∆B141 (101,396-466,499), ∆B163 (451,557-651,863), ∆B180 (635,940-869,642), ∆B181 (870,505-1,129,758), ∆B108 (1,131,168-1,169,073), ∆B109 (1,180,566-1,204,770), ∆B179 (1,207,052-1,322,226), ∆B118 (1,323,078-1,528,150), ∆B124 (1,529,711-1,677,882), and ∆B161 (pSymB nt. 1,679,723-49,523) (Figure S1). In the case of the ∆B154, ∆B163, ∆B181, ∆B108, ∆B118, ∆B124, and ∆B161 deletions, the deletions were recombined via transduction of neomycin resistance into each of the fusion library strains of interest. In the case of the ∆B141, ∆B180, and ∆B109 deletions, the gene fusions of interest were recombined via transduction of gentamicin resistance into each deletion mutant strain. For the ∆B179 deletion, the gene fusions of interest were first recombined via transduction into *S. meliloti* RmP2719, which carries the essential *engA* and tRNA^arg^ genes integrated into the chromosome (diCenzo *et al.* 2013), following which the ∆B179 deletion was recombined via transduction of neomycin resistance into the resulting strains.

The deletions ∆B110 (1,207,052-1,255,032), ∆B116 (1,256,503-1,307,752), and ∆B158 (1,308,912-1,322,226) were combined with the *smc03121*::*lacZ* gene fusion using the method described for ∆B179 above. Four new pSymB deletion mutants were constructed as follows. The plasmid pTH1943 (Milunovic *et al.* 2014), containing a *FRT* (Flippase Recognition Target) site, was conjugated into four fusion library strains (SmFL2626, SmFL2987, SmFL4163, SmFL4484) that each contained a *FRT* site (Cowie *et al.* 2006), and neomycin resistant transconjugants were purified. The plasmid pTH2505 (Zhang *et al.* 2012), which expresses the Flp recombinase, was conjugated to each of the four transconjugants resulting in the deletion of the region flanked by the *FRT* sites. This produced the deletions ∆B182 through ∆B185. Each deletion was then combined with the *smc03121*::*lacZ* gene fusion using the method described for ∆B179 above.

### Construction of single gene knockout mutants

Fragments internal to the following genes were PCR amplified and introduced into *Spe*I digested pTH1937 (Milunovic *et al.* 2014): *bdhA* (oligo: DF129/DF130), *xdhA2* (DF131/DF132), *xdhB2* (DF133/DF134), *smb20847* (DF135/DF136), *smb20848* (DF137/DF138), *guaD2* (DF139/DF140), *lldD1* (DF141/DF142), and *smb20851* (DF143/DF144). Plasmids were conjugated to *S. meliloti* RmP3757, which contains the *smc03121*::*lacZ* gene fusion and the essential *engA* and tRNA^arg^ pSymB genes integrated into the chromosome. To construct the *bdhA phbB* double mutant, the *bdhA*::pTH1937 allele was first transduced into the SmFL4407 (*smc03121*::*lacZ*) fusion library strain followed by transduction of the *phbB*::ΩSp^R^ allele from Rm11347 (Aneja *et al.* 2004).

### Data availability

All strains are available upon request. Gene expression data are available at GEO with the accession number GSE106129. File S1 contains detailed descriptions of all supplemental files. File S2 contains the processed RNA-seq data for chromosomal genes. File S3 contains the processed RNA-seq data for pSymB genes. File S4 contains the processed RNA-seq data for pSymA genes. File S5 contains the predicted operons in the *S. meliloti* genome. Table S1 contains strain and plasmid descriptions. Table S2 contains oligonucleotide sequences. Table S3 contains gene pangenome classifications. Table S4 lists the best putative pSymA/pSymB encoded homolog of chromosomal genes. Table S5 contains KEGG, GO, and KEGG functional enrichment data for chromosomal genes. Figure S1 displays the location of pSymB deletions. Figure S2 contains growth data for the strains used in the RNA-sequencing experiment.

## Results

### Global transcriptional consequences of pSymA and pSymB removal

As described in Table 1, the *S. meliloti* wild type strain and the ∆pSymA, ∆pSymB, and ∆pSymAB strains each contain a different set of the three DNA replicons. The presence of the chromosome is the only consistent feature of these four strains, and previous whole genome sequencing confirmed that the chromosome of these strains is highly isogenic, with only four polymorphisms detected (diCenzo *et al.* 2016b). Thus, differences in the phenotypes of these strains can be ascribed to the presence/absence of pSymA or pSymB with high confidence, and are unlikely due to chromosomal polymorphisms.

An RNA-sequencing experiment was performed to examine how the loss of one or more replicon influences the transcriptional pattern of the remaining replicons. RNA was isolated from the above four strains grown to mid-exponential phase (OD_600_ ∼0.7) in minimal M9-sucrose medium. In the wild type pSymA^+^ pSymB^+^ strain, approximately 84% of sequencing reads mapped to the chromosome, 4% to pSymA, and 12% to pSymB. Considering replicon sizes, chromosomal transcripts were ∼1.5 fold over-represented in the transcriptome, while pSymB and pSymA were ∼1.9 fold and ∼5 fold under-represented, respectively. This highlights the varying levels of importance of each replicon to the laboratory growth of *S. meliloti*.

**Figure 1:**
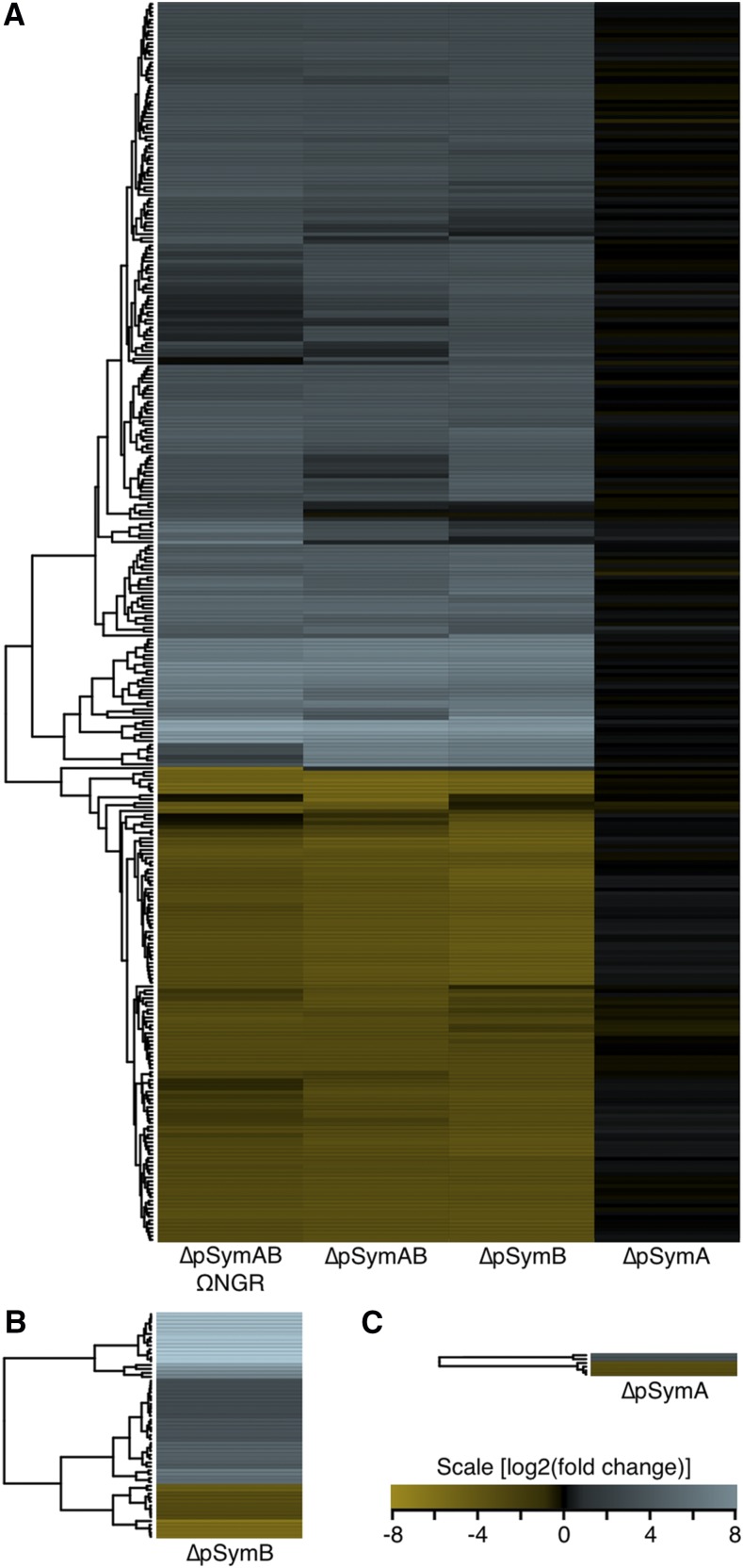
Overview of the transcriptional consequences of replicon removal. Heatmaps summarizing the expression of all genes differentially expressed (≥ twofold, p-value ≤ 0.01) in at least one strain are shown. (A) The 290 chromosomal genes and 25 chromosomal ncRNAs, (B) the 58 pSymA genes, (C) and the six pSymB genes differentially expressed are shown. Expression of all genes are shown relative to that of the wild type *S. meliloti* strain RmP3499 (pSymA^+^ pSymB^+^). Those in blue are up-regulated compared to wild type while those in yellow are down-regulated compared to wild type. Expression changes are displayed on a log_2_ scale.

**Figure 2:**
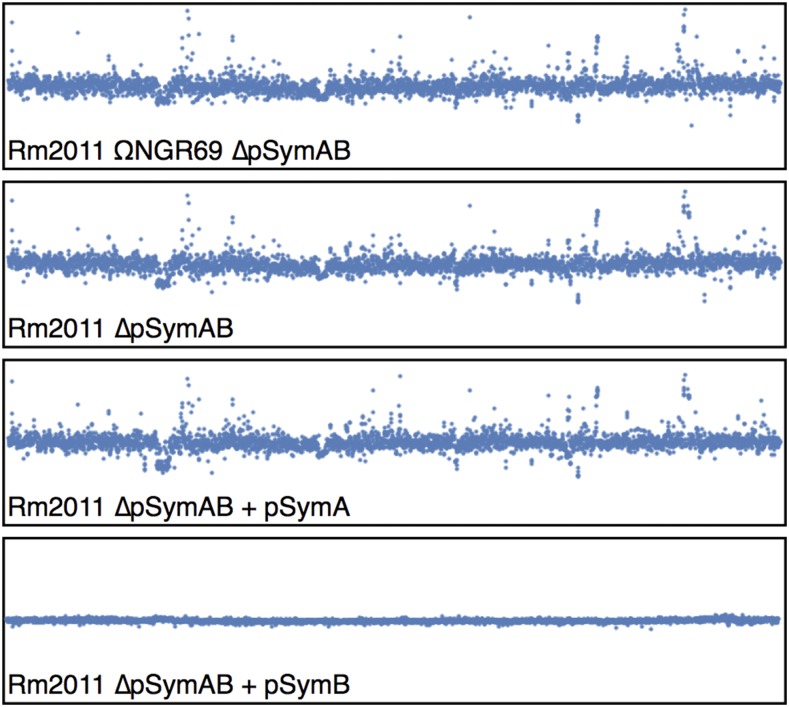
Chromosomal localization of the differentially expressed genes. Each box displays the expression of all 3824 genes and ncRNAs on the *S. meliloti* chromosome, with the genes displayed in the same order they are situated on the chromosome. Each dot represents a single gene, and displays the expression of the gene relative to that of the wild type *S. meliloti* strain RmP3499 (pSymA^+^ pSymB^+^). Expression changes are displayed on a log_2_ scale, with the top and bottom of each box representing a 64-fold up-regulation or down-regulation relative to wild type.

The removal of the pSymA megaplasmid (∼1,300 genes) had little impact on the expression of the remaining transcripts (Figures 1 and 2); no chromosomal transcripts displayed a statistically significant change in expression (p-value ≤ 0.01, fold change ≥ 2), while only six genes from four transcripts were differentially regulated on pSymB (two genes up-regulated, four genes down-regulated). This was in stark contrast to the large effect observed when the pSymB chromid (∼1,600 genes) was absent (Figures 1, 2). A total of 25 chromosomal ncRNAs (22 up-regulated, 3 down-regulated) and 271 chromosomal genes (157 up-regulated, 114 down-regulated) from 172 transcripts were differentially expressed in at least one of ∆pSymB or ∆pSymAB compared to wild type, accounting for nearly 8% of all chromosomal genes. An additional 58 pSymA encoded genes (44 up-regulated, 14 down-regulated, ∼4.4% of pSymA genes) from 35 transcripts were differentially expressed when pSymB was absent (Figure 1). Of the 296 chromosomal genes/ncRNAs differentially regulated in either RmP3497 or RmP3496, only 18 genes and 3 ncRNAs displayed ≥ twofold change in one strain and < 1.5 fold change in the other, suggestive of little combinatorial effect from the removal of pSymA together with pSymB. Overall, these data suggested that, in the tested conditions, the evolutionarily older pSymB chromid was more transcriptionally integrated with the chromosome than was the evolutionarily younger pSymA megaplasmid.

**Figure 3:**
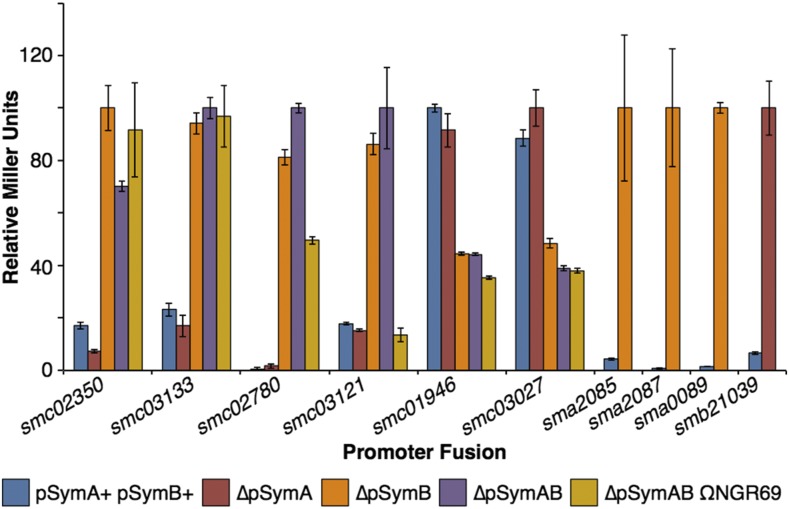
Reporter gene fusion confirmation of the RNA-seq data. Transcriptional fusions of genes of interest to either a *lacZ* or *gusA* reporter gene were prepared in each strain used for the RNA-seq analysis. The β-galactosidase or β-glucuronidase activities for each fusion, expressed as relative Miller Units, are shown. For each fusion, the values are given relative to the strain with the highest activity, and the strain with the highest activity was normalized to a value of 100. Data points represent the means of triplicate samples, with the error bars indicating the standard error.

Reporter gene fusions to 10 promoters that were differentially expressed in the absence of pSymA and/or pSymB were identified in the *S. meliloti* fusion library collection (Cowie *et al.* 2006) and introduced into each strain to validate the RNA-seq data. These ten promoters were chosen as they either represented different expression patterns, displayed large differences in expression between strains, or we considered the genes to be particularly interesting. For all 10 of the promoter fusions tested, the results of the enzyme assays were consistent with the transcriptional patterns detected in the RNA-seq dataset (Figure 3), confirming that the RNA-seq data accurately captured the transcriptional consequences of the massive genome reductions.

### Inter-replicon gene flow contributes to increased cross-replicon integration

We previously reported that an inter-replicon translocation event resulted in the transfer of a 69 kilobase region (66 genes), the ETR region, from the chromosome to the pSymB chromid in a *S. meliloti* ancestor (diCenzo *et al.* 2013). We have since integrated the corresponding ETR region from *S. fredii* NGR234 into the *S. meliloti* chromosome and produced a derivative lacking pSymA and pSymB (∆pSymAB_ΩNGR69_; Table 1) (diCenzo *et al.* 2016b). To test how the loss of this “chromosomal” gene region influenced transcription, RNA-seq was used to compare the transcriptome of ∆pSymAB_ΩNGR69_ with ∆pSymAB, ∆pSymB, ∆pSymA, and wild type (Figures 1, 2). Mapping the sequencing reads to the *S. fredii* NGR234 genome confirmed that the genes of the ETR region in the ∆pSymAB_ΩNGR69_ genome were expressed (not shown).

The up-regulation of seven genes from two transcripts (*smc03116-smc03121*, and *smc04246*) in the absence of pSymB could be traced primarily to the loss of the ETR region as these genes were expressed two to sixfold higher in ∆pSymAB than in ∆pSymAB_ΩNGR69_ (File S2; see *smc03121* in Figure 3 for an example). Several additional genes displayed an intermediate phenotype in ∆pSymAB_ΩNGR69_ compared to ∆pSymAB and the wild type, suggesting the phenotype was partially linked to the loss of the ETR region. Eight genes from five transcripts were up-regulated compared to wild type primarily when the ETR region was present but the rest of pSymB was absent; these genes were expressed ∼ two to ninefold higher in ∆pSymAB_ΩNGR69_ than in ∆pSymAB. Finally, one gene (*smc03107*) was down-regulated compared to wild type only in the absence of pSymB if the ETR region was present. These data revealed that the flow of genes between replicons in the *S. meliloti* genome contributed to their transcriptional integration. Additionally, these data illustrated how genomic context influenced the transcriptional consequences of gene deletion/addition.

### Causes of transcriptional changes can be traced to specific regions of pSymB

**Figure 4:**
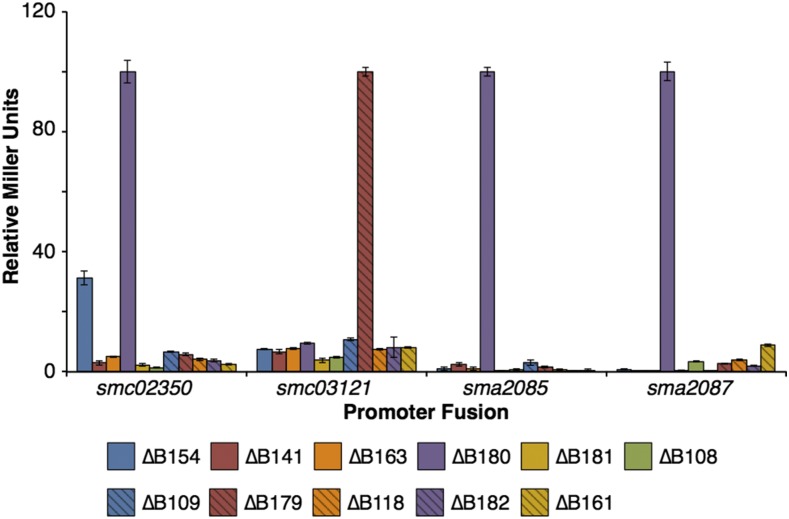
Localization of pSymB regions that influence expression of chromosome or pSymA genes. Transcriptional *lacZ* or *gusA* reporter gene fusions to genes of interest recombined into the same strain as each of the eleven large-scale pSymB deletion that cumulatively span ∼98% of pSymB. The β-galactosidase or β-glucuronidase activities for each fusion, expressed as relative Miller Units, are shown. For each fusion, the values are given relative to the strain with the highest activity, and the strain with the highest activity was normalized to a value of 100. The location of the deletions are shown in Figure S1. Data points represent the means of triplicate samples, with the error bars indicating the standard error.

It was possible that the observed transcriptional changes were a consequence of the reduced growth rate of strains lacking pSymB instead of a direct consequence of loss of specific pSymB encoded genes; in the tested medium, the generation time of strains lacking pSymB was ∼5.3 hr compared to ∼3.1 hr for the wild type (Figure S2). Therefore, expression from four promoters up-regulated in the absence of pSymB was examined in a collection of 11 large-scale pSymB deletion mutants that cumulatively remove ∼98% of pSymB (Figure S1) (Milunovic *et al.* 2014); these deletion mutants displayed little growth rate difference compared to the wild type (see well D11 of plate 1 of the Biolog Phenotype MicroArray data of diCenzo *et al.* 2016a). Expression from the divergently transcribed promoters of the five gene *sma2085-sma2077* and eight gene *sma2087-sma2101* operons on pSymA were observed to be up-regulated specifically in the ∆B180 (pSymB nt. 635,940-869,642) deletion (Figure 4). Expression of the chromosomal promoter of the six gene *smc0235-smc02344* operon was up-regulated in both the ∆B180 and ∆B154 (pSymB nt. 62,137-100,636) deletions (Figure 4). Finally, the six gene *smc03121-smc03116* operon was specifically up-regulated in the ∆B179 (pSymB nt. 1,207,052-1,322,226) deletion (Figure 4). Hence, while the expression of an unknown number of genes may have simply been a consequence of differences in growth rates, these observations confirmed that several of the transcriptional changes were directly attributable to the loss of specific pSymB encoded genes.

### Few detectable biases in chromosomal genes up-regulated in the absence of pSymB

We attempted to determine whether there was any feature that separated chromosomal genes up-regulated in the absence of pSymB from the average chromosomal gene. As can be seen in Figure 2, genes up-regulated upon removal of pSymB were distributed across the entire chromosome with no obvious locational bias. In order to limit the number of false positives, only the 146 genes up-regulated (≥twofold) in at least two of the three strains lacking pSymB were considered for the subsequent analyses. No statistically significant biases were observed in the pangenome classification (core, accessory, unique) of genes up-regulated in the absence of pSymB compared to genes whose expression was unchanged or down-regulated (Table S3).

Greater than 10% of *S. meliloti* chromosomal genes may have a functionally redundant copy on either pSymA or pSymB (diCenzo and Finan 2015), and we therefore wondered if the up-regulation was a result of loss of pSymB encoded homologs. A Blast bidirectional best hit approach was used to predict putative functional homologs between chromosomal genes and genes on pSymA/pSymB (see Materials and Methods). It was observed that of the genes whose expression did not change across strains, ∼5% had a best putative functional homolog on pSymA and an additional ∼5% had a best putative functional homolog on pSymB (Table S4). Of the genes up-regulated in the absence of pSymB, ∼5% had a best putative functional homolog on pSymA, whereas ∼15% had a best putative functional homolog on pSymB (Table S4). This therefore supported that some of genes up-regulated in the absence of pSymB may have been an attempt to compensate for the loss of a functional homolog, although the majority of changes could not be explained by functional redundancy.

Genes were annotated with COG terms, GO terms, and KEGG pathway terms in an attempt to identify functional biases between genes up-regulated or not up-regulated upon loss of pSymB (Table S5). The only biases observed were in the broad COG categories of inorganic ion transport and metabolism (COG P), transcription (COG K), and energy production and conversion (COG C). No biases were observed in GO term or KEGG pathway annotations. Hence, no obvious functional role separated the genes up-regulated when pSymB was removed compared to the average chromosomal gene.

### Cell motility is decreased in the absence of pSymB

Genes down-regulated in the absence of pSymB are spread across the chromosome; however, a clear cluster of down-regulated genes was evident (Figure 2). This cluster contained flagellum related genes. When a functional annotation (COG, GO, and KEGG pathways) of the 90 genes down-regulated (≤ twofold) in at least two strains lacking pSymB was performed, a clear enrichment was observed in sensory transduction, motility, and chemotaxis related processes (Table S5). Down-regulation of the ten gene *flgB-fliP* operon was confirmed using a transcriptional gene fusions (fusion *smc03027* in Figure 3). These results therefore indicated that the loss of pSymB results in decreased expression of taxis related genes in the tested medium. Indeed, in earlier experiments we observed that strains lacking pSymB displayed reduced swimming motility in TY soft agar plates (data not shown), although we could not rule out that this was simply a consequence of the reduced growth rate.

### Loss of bdhA results in smc03121 up-regulation through 3-hydroxybutyrate accumulation

**Figure 5:**
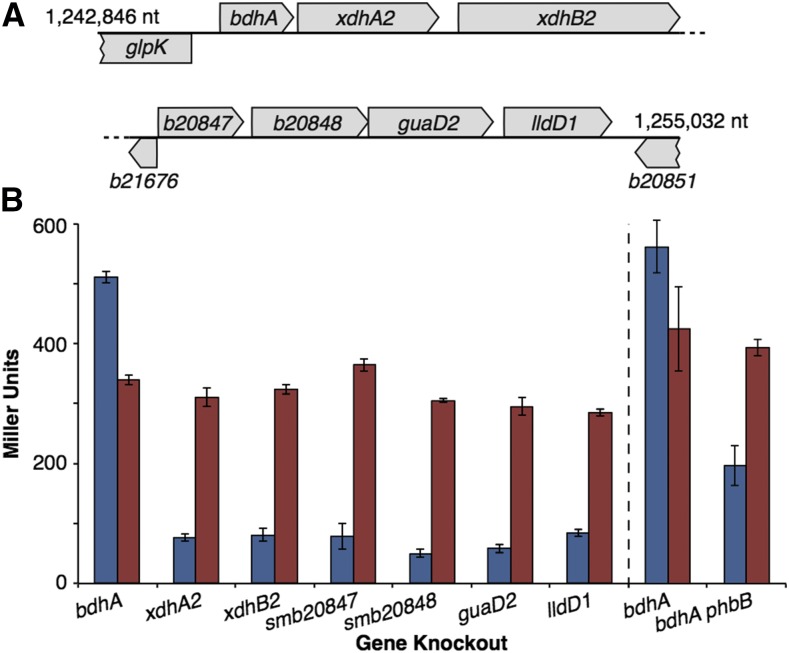
Effect of gene knockouts and 3-hydroxybutyrate on *smc03131* expression. (A) Schematic of the gene organization of the 12 kb region whose deletion resulted in induction of *smc03121*. (B) β-Galactosidase activity from a *smc03121*::*lacZ* fusion in either M9-sucrose minimal medium (blue) or M9-sucrose supplemented with 10 mM 3-hydroxybutyrate (red). The gene(s) mutated in each strain are indicated along the x-axis, and expression values are presented as Miller units. The vertical dashed line separates assays that were performed in independent experiments. Values represent the mean of triplicate samples, with the error bars indicating the standard error.

The data presented earlier indicated that the *smc03121* operon was transcriptionally up-regulated in the ∆pSymB and ∆pSymAB strains, that this phenotype could be traced to the B179 region of pSymB that includes the ETR region (Figure 4), and that re-introduction of just the ETR region was sufficient to restore wild type levels of *smc03121* expression (Figure 3). Using a series of sub-deletions within the B179 region, the responsible locus within the B179 region was narrowed down to a 12 kb region (pSymB nt. 1,242,846-1,255,032) spanning eight complete genes (data not shown; Figure 5A). Single cross-over plasmid integration was used to construct knockouts of seven of these eight genes in a wild type background containing a *smc03121*::*lacZ* fusion (Figure 5A), and it was observed that disrupting just the *bdhA* gene, encoding a 3-hydroxybutyrate dehydrogenase (Aneja and Charles 1999), resulted in transcriptional up-regulation of *smc03121* (Figure 5B). This result was also seen in a recent RNA-seq study of *S. meliloti* poly-3-hydroxybutyrate (PHB) cycle mutants (D’Alessio *et al.* 2017). However, *smc03121* was induced and expressed equally in all eight gene mutants when the medium was supplemented with 10 mM 3-hydroxybutyrate (Figure 5B). We therefore reasoned that 3-hydroxybutyrate may accumulate, and be secreted into the medium, in a *bdhA* null mutant, resulting in the observed *smc03121* up-regulation. As the sole source of 3-hydroxybutyrate in the *S. meliloti* iGD1575 *in silico* metabolic reconstruction is from PHB breakdown (diCenzo *et al.* 2016a), a *phbB*::ΩSp mutation was introduced into a *smc03121*::*lacZ bdhA*::pTH1937 strain as this mutation prevents PHB synthesis (Aneja *et al.* 2004). Indeed, expression of *smc03121* was lower in the *bdhA phbB* double mutant than in the *bdhA* single mutant when grown in M9-sucrose minimal medium lacking 3-hydroxybutyrate (Figure 5B). Hence, it was concluded that, like in *Ralstonia eutrophus* (Doi *et al.* 1990; Taidi *et al.* 1995), continual PHB synthesis and degradation was occurring during exponential growth in M9-sucrose, and that mutation of *bdhA* resulted in the accumulation and export of the BdhA substrate, 3-hydroxybutyrate, thereby resulting in the induction of the *smc03121-smc03116* transport and metabolic operon.

## Discussion

The *S. meliloti* genome is a good model for the study of the multipartite genome as it contains three replicons each with distinct characteristics. In particular, the pSymA megaplasmid is derived primarily through recent and ongoing HGT whereas pSymB is a more stable replicon derived largely through ancient HGT (Galardini *et al.* 2013). Here, RNA-sequencing was used to obtain a global overview of the transcriptional consequences of the removal of these large secondary replicons from the genome to gain insights into the level of transcriptional integration of these replicons and the influence of the age of the replicon. Importantly, it was possible to link transcriptional changes to the loss of specific gene regions. This confirmed that several of the observed expression changes are not simply indirect consequences of the reduced growth rate, but instead reflect regulatory or metabolic interactions between the replicons.

The absence of pSymB resulted in major transcriptome changes with ∼8% of chromosomal genes and ∼4% of pSymA genes differing in expression (Figure 1). This was consistent with metabolomics work that identified large perturbations in the metabolome of strains lacking pSymB (Fei *et al.* 2016). In contrast, the data reported here indicated that removal of pSymA impacted expression of a mere four operons (Figure 1). This was similar to previous 2D gel electrophoretic work that detected only 30 chromosome or pSymB encoded proteins that differed in abundance following pSymA removal (Chen *et al.* 2000), and metabolomics work that observed few metabolic differences between strains with and without pSymA (Fei *et al.* 2016). However, transcriptional integration of pSymA with the chromosome may still occur during specialized processes; for example, the pSymA-encoded symbiotic transcriptional regulator FixJ has chromosomally encoded target genes (Bobik *et al.* 2006). Overall, these studies support that newly acquired replicons are poorly integrated into the central transcriptional and metabolic networks of the cell, and that continual co-existence of two replicons results in a gradual inter-dependency of the replicons at multiple levels.

The analyses described here allowed an examination of how secondary replicon removal influences chromosomal gene expression, but they did not allow study of how transcription of secondary replicons is influenced by the primary replicon. However, the effects of pSymB removal *vs.* pSymA removal on the transcription of genes on the other replicon can serve as a proxy since pSymB has several chromosome-like features and was already present at the time pSymA was acquired. Whereas the loss of pSymB influenced expression of ∼4% of pSymA genes, less than 0.5% of pSymB genes were impacted by the loss of pSymA (Figure 1). Previously, an *in silico* analysis predicted that approximately twofold more genes on pSymA or pSymB are regulated by chromosomal transcription factors than chromosomal genes regulated by pSymA or pSymB encoded transcription factors (Galardini *et al.* 2015). Together, these results suggest that existing replicons ‘domesticate’ newly acquired replicons; *i.e.*, there is a higher likelihood of genes on newly acquired replicons being integrated into existing regulatory networks than there is of existing genes being transcriptionally modified in response to acquisition of the new replicon.

Plasmids and other secondary replicons often have specialized functions, and have been postulated to play a key role in adaptation and evolution (Reanney 1976). A transcriptional separation from chromosomal genes may help facilitate successful horizontal transfer of the replicon by reducing the occurrence of off-target effects. Selection may therefore have favored the spread of replicons lacking significant ties to chromosomal elements, potentially explaining, in part, the low level of integration of pSymA into the central cellular networks. Thus the level of integration of two replicons in a genome may be a function of selection for improved horizontal transfer efficiency and the length of replicon co-evolution. However, whether or not increased integration in central networks only occurs after the replicon has largely lost the ability to undergo horizontal transfer requires further study.

The transcriptional inter-dependence of each replicon in a genome can be mediated through multiple mechanisms. The simplest explanation is that genes are integrated into the regulons of transcription factors located on other replicons as has been predicted *in silico* (Galardini *et al.* 2015). Another possibility is through the transfer of genes between replicons. It was shown that the transcriptional changes of seven chromosomal genes following the removal of pSymB were dependent on the loss of the ETR region that translocated to pSymB in a recent *S. meliloti* ancestor (Figures 1, 3) (diCenzo *et al.* 2013; diCenzo *et al.* 2016b). Functional redundancies can have an influence as well. Maintenance of functional redundancy can be driven by reductions in expression of the duplicate genes (Qian *et al.* 2010), and deletion of one of the gene could therefore be complemented by induction of the other. The data presented here supports this notion as genes with putative homologs on pSymB are over-represented in the gene set that is up-regulated following pSymB removal. These include several ABC-type transport systems and cytochrome proteins (Table S4, File S2). Moreover, the transcriptional changes could be indirect responses mediated through shifts in the metabolic profile of the cell or environment. This was exemplified by the *smc03131* operon, whose induction by the removal of pSymB appeared to be a consequence of an accumulation of 3-hydroxybutyrate due to an inability of strains lacking pSymB to degrade the 3-hydroxybutyrate produced through PHB breakdown (Figure 5).

Considering that pSymA and pSymB were formed largely through either recent or ancient HGT, respectively, the data presented here can be interpreted more broadly with respect to HGT. In particular, this data may suggest that a gene acquired through recent HGT is more likely to be maintained in the genome if it does not immediately disturb the existing central transcriptional network, with transcriptional integration being a gradual process that occurs following the gene’s acquisition. In support of this, transcriptional regulators acquired through HGT primarily regulate genes that are co-transferred with the regulator whereas global regulators are primarily vertically transmitted (Price *et al.* 2008). Previous work has also indicated that recently acquired genes are regulated by few transcription factors and that it takes millions of years for regulation to reach levels similar to that of native genes (Lercher and Pál 2008). Moreover, horizontally acquired genes often encode functions located at the periphery of metabolic networks (Pál, Papp, and Lercher 2005a), and genes most likely to be maintained following their acquisition generally have low rates of transcription or translation (Taoka *et al.* 2004; Park and Zhang 2012).

We produced the genome reduced strains used here in order to study the genome biology and evolution of *S. meliloti* as well as the contributions of these replicons to nitrogen fixing symbiosis (diCenzo *et al.* 2014; diCenzo *et al.* 2016b). This work completes our systems-level analyses of these strains, which has included phenomics (diCenzo *et al.* 2014), metabolomics (Fei *et al.* 2016), and now transcriptomics. The ∆pSymAB strain represents a genome reduction of nearly 2,900 genes and displays many phenotypic, metabolomic, and transcriptomic perturbations. As shown by the work with *smc03131* and *bhdA* in this study (Figure 5), these data sets together with the existing *S. meliloti* genomic resources, including the fusion and deletion libraries (Cowie *et al.* 2006; Milunovic *et al.* 2014), can facilitate rapid linkage of phenotypes to the loss of specific genes. As such, we expect future mining of these data to reveal many interesting insights into the genetics of *S. meliloti*.

## Supplementary Material

Supplemental Material is available online at www.g3journal.org/lookup/suppl/doi:10.1534/g3.117.300405/-/DC1.
