# Supplementary material for: Inter-replicon Gene Flow Contributes to Transcriptional Integration in the Sinorhizobium meliloti Multipartite Genome
Source: G3 (Bethesda). 2018 Mar 21;8(5):1711–20. doi: 10.1534/g3.117.300405 (PMC5940162; doi:10.1534/g3.117.300405)
Supplement: Supplementary file 1 [file 1711FigureS1.pdf]

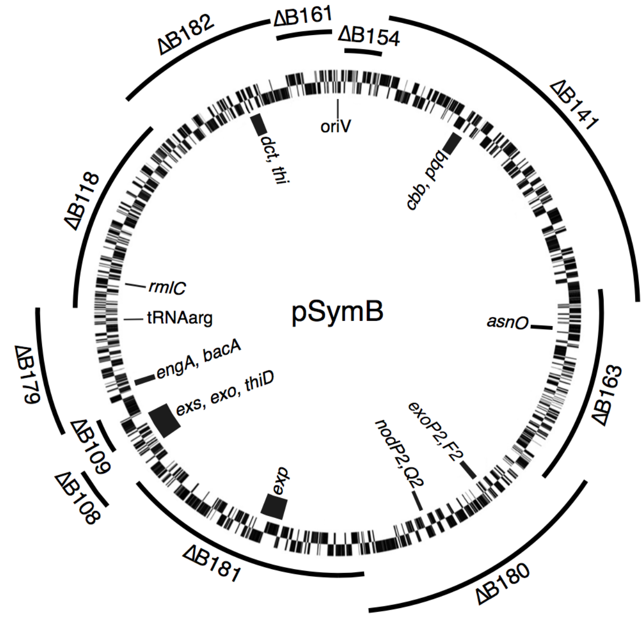

Strain	Deleted region (nucleotide position)	Strain	Deleted region (nucleotide position)
ΔB154	62,137-100,636	ΔB154	1,180,466-1,204,770
ΔB141	101,396-466,499	ΔB141	1,207,052-1,322,226
ΔB163	451,557-651,863	ΔB163	1,323,078-1,528,150
ΔB180	635,940-869,642	ΔB180	1,529,711-1,677,882
ΔB181	870,505-1,129,758	ΔB181	1,679,723-49,523
ΔB108	1,131,168-1,169,073		

Figure S1. Deletion library mutants used in the localization of the regions whose loss influenced expression of chromosomal genes. A schematic representation of pSymB and the location of the deletions employed in this study. The inner circle represents pSymB with annotated genes shown by the individual lines. The outer curves indicate the region of pSymB that has been removed in the corresponding deletion mutant. Several notable loci are indicated along the inner circle for reference. *dct*: *dctA,B,D. thi*: *thiC,O,G,E. exs*: *exsA-I. exo*: *exoA,B,F1,H,I,K-Q,T-Z. exp*: *wgeA-H, wgdA,B, wggR, wgcA, wgaA,B,D-J. cbb*: *cbbA,F,L,P,R,S,T,X. pqq*: *pqqA-E.*
