# Supplementary figures and images for: Inter-replicon Gene Flow Contributes to Transcriptional Integration in the Sinorhizobium meliloti Multipartite Genome
Source: G3 (Bethesda). 2018 Mar 21;8(5):1711–20. doi: 10.1534/g3.117.300405 (PMC5940162; doi:10.1534/g3.117.300405)

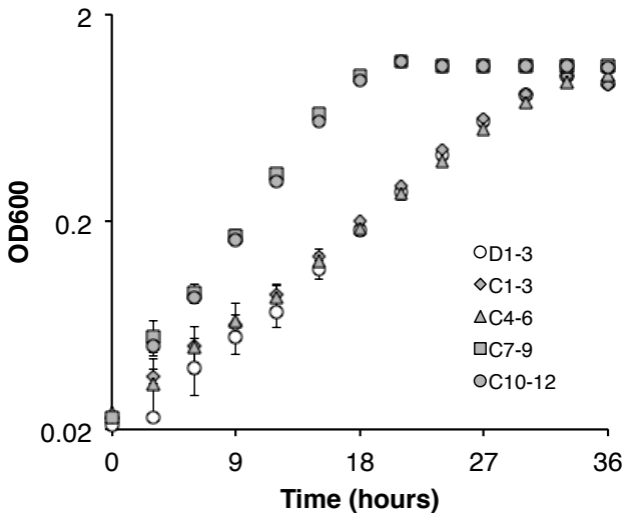

● pSymA+ pSymB+  
3.13 ± 0.11

■ ΔpSymA  
3.12 ± 0.14

△ ΔpSymB  
5.57 ± 0.33

◆ ΔpSymAB  
5.24 ± 0.21

○ ΔpSymAB ΩNGR69  
5.00 ± 0.24

Supplement: Supplementary file 2 [file 1711FigureS2.pdf]
