# Supplementary material for: Inter-replicon Gene Flow Contributes to Transcriptional Integration in the Sinorhizobium meliloti Multipartite Genome
Source: G3 (Bethesda). 2018 Mar 21;8(5):1711–20. doi: 10.1534/g3.117.300405 (PMC5940162; doi:10.1534/g3.117.300405)
Supplement: Supplementary file 8 [file 1711FileS1.pdf]

File S1. Contains detail descriptions of all supplemental files.

File S2. RNA-seq data for chromosomal genes. This file contains the analyzed RNA-seq dataset for chromosomal protein coding genes and ncRNAs. It includes four worksheets. The 'All data' sheet includes the data for all chromosomal elements, and presents the gene name, the gene identifier, the predicted protein function, the fold change relative to *S. meliloti* RmP3499 (pSymA$^+$pSymB$^+$) on a $\log_2$ scale, and the adjusted p-value. An empty cell for the fold change indicates no mapped reads, while an empty cell for the adjusted p-value indicates a value greater than 1. The 'Higher expression' sheet includes the same data but only for the genes that display higher expression in at least one strain relative to RmP3499 (p-value ≤ 0.01, fold change ≥ 2). Genes in this sheet are grouped based on the strains in which their expression is elevated. The 'Lower expression' sheet includes the same data but only for the genes that display lower expression in at least one strain relative to RmP3499 (p-value ≤ 0.01, fold change ≤ 0.5). Genes in this sheet are grouped based on the strains in which their expression is diminished. The 'Legend' sheet provides a description of the groupings present in the previous two sheets, and a description of the strains.

File S3. RNA-seq data for pSymB genes. This file contains the analyzed RNA-seq dataset for pSymB protein coding genes and ncRNAs. It includes three worksheets. The 'All data' sheet includes the data for all pSymB elements, and presents the gene name, the gene identifier, the predicted protein function, the gene expression fold change in *S. meliloti* RmP3498 (ΔpSymA) relative to *S. meliloti* RmP3499 (pSymA$^+$pSymB$^+$) on a $\log_2$ scale, and the adjusted p-value. An empty cell for the fold change indicates no mapped reads, while an empty cell for the adjusted p-value indicates a value greater than 1. The 'Higher expression' sheet includes the same data but only for the genes that display higher expression in RmP3498 relative to RmP3499 (p-value ≤ 0.01, fold change ≥ 2). The 'Lower expression' sheet includes the same data but only for the genes that display lower expression in RmP3498 relative to RmP3499 (p-value ≤ 0.01, fold change ≤ 0.5).

File S4. RNA-seq data for pSymA genes. This file contains the analyzed RNA-seq dataset for pSymA protein coding genes and ncRNAs. It includes three worksheets. The 'All data' sheet includes the data for all pSymA elements, and presents the gene name, the gene identifier, the predicted protein function, the gene expression fold change in *S. meliloti* RmP3497 (ΔpSymB) relative to *S. meliloti* RmP3499 (pSymA$^+$pSymB$^+$) on a $\log_2$ scale, and the adjusted p-value. An empty cell for the fold change indicates no mapped reads, while an empty cell for the adjusted p-value indicates a value greater than 1. The 'Higher expression' sheet includes the same data but only for the genes that display higher expression in RmP3497 relative to RmP3499 (p-value ≤ 0.01, fold change ≥ 2). The 'Lower expression' sheet includes the same data but only for the genes that display lower expression in RmP3497 relative to RmP3499 (p-value ≤ 0.01, fold change ≤ 0.5).

File S5. *S. meliloti* operon prediction. Operons were predicted in the *S. meliloti* genome based on the RNA-seq data generated in this study using the Rockhopper software. This file lists the predicted start and stop nucleotides for each operon, the strand on which the operon is located, the number of genes in the operon, and which genes belong to the operon. If a gene of interest is not present in this file, then the gene was predicted to not belong to an operon.

Table S1. Bacterial strains and plasmids. A description of all bacterial strains and plasmids used in this work.

Table S2. Oligonucleotide used in this study. The sequence of all oligonucleotide primers used in this work.

Table S3. Pangenome classification of chromosomal *S. meliloti* genes. Classification of all chromosomal genes, as well as just those either up-regulated or down-regulated following removal of pSymB, as part of the 'core genome', 'accessory genome', or 'unique genome' of *S. meliloti*.

Table S4. Chromosomally encoded proteins with a putative pSymA or pSymB encoded homolog. This table lists the 480 chromosomally encoded proteins with at least one putative homolog encoded by pSymA or pSymB. The chromosomally encoded protein is indicated as is the best scoring putative homolog, and the table also lists the annotated function of each protein, the percent of the proteins aligned, the percent identity, the E-value of the blast result, and whether expression of the corresponding chromosomal gene is higher, unchanged, or lower in strains lacking pSymB. A description of each column of the table is included in the file.

Table S5. Functional analyses of *S. meliloti* chromosomal genes. COG, GO, and KEGG functional enrichment analysis of all chromosomal genes, as well as just those either up-regulated or down-regulated following removal of pSymB.

Figure S1. Deletion library mutants used in the localization of the regions whose loss influenced expression of chromosomal genes. A schematic representation of pSymB and the location of the deletions employed in this study. The innter circle represents pSymB with annotated genes shown by the individual lines. The outer curves indicate the region of pSymB that has been removed in the corresponding deletion mutant. Several notable loci are indicated along the inner circle for reference. *dct*: *dctA,B,D. thi*: *thiC,O,G,E. exs*: *exsA-I. exo*: *exoA,B,F1,H,I,K-Q,T-Z. exp*: *wgeA-H, wgdA,B, wggR, wgcA, wgaA,B,D-J. cbb*: *cbbA,F,L,P,R,S,T,X. pqq*: *pqqA-E.*

Figure S2. Growth curves of the strains used in the RNA-seq experiment. Growth of each strain was monitored in M9-sucrose medium in 150 µL cultures. OD_{600} values are not corrected for pathlength. Data points represent the average of triplicate cultures, and the error bars indicate the standard deviation. A figure key is provided at the bottom of the figure; below each strain name, the average generation time ± standard deviation is provided.
